# Continuous assessment of neuro-ventilatory drive during 12 h of pressure support ventilation in critically ill patients

**DOI:** 10.1186/s13054-020-03357-9

**Published:** 2020-11-20

**Authors:** Rosa Di mussi, Savino Spadaro, Carlo Alberto Volta, Nicola Bartolomeo, Paolo Trerotoli, Francesco Staffieri, Luigi Pisani, Rachele Iannuzziello, Lidia Dalfino, Francesco Murgolo, Salvatore Grasso

**Affiliations:** 1 Dipartimento dell’Emergenza e Trapianti d’Organo (DETO), Sezione di Anestesiologia e Rianimazione, Università degli Studi di Bari “Aldo Moro”, Ospedale Policlinico, Piazza Giulio Cesare 11, Bari, Italy; 2 Dipartimento di Morfologia, Chirurgia e Medicina Sperimentale, Sezione di Anestesiologia e Terapia Intensiva Universitaria, Università degli Studi di Ferrara, Ferrara, Italy; 3 Dipartimento di Scienze Biomediche ed Oncologia Umana, Cattedra di Statistica Medica, Università degli Studi Aldo Moro, Bari, Italy; 4 Dipartimento dell’Emergenza e Trapianti d’Organo (DETO), Sezione di Chirurgia Veterinaria, Università degli Studi di Bari “Aldo Moro”, Bari, Italy

**Keywords:** Mechanical ventilation, Assisted modes of ventilation, Pressure support ventilation (PSV)

## Abstract

**Introduction:**

Pressure support ventilation (PSV) should allow spontaneous breathing with a “normal” neuro-ventilatory drive. Low neuro-ventilatory drive puts the patient at risk of diaphragmatic atrophy while high neuro-ventilatory drive may causes dyspnea and patient self-inflicted lung injury. We continuously assessed for 12 h the electrical activity of the diaphragm (EAdi), a close surrogate of neuro-ventilatory drive, during PSV. Our aim was to document the EAdi trend and the occurrence of periods of “Low” and/or “High” neuro-ventilatory drive during clinical application of PSV.

**Method:**

In 16 critically ill patients ventilated in the PSV mode for clinical reasons, inspiratory peak EAdi peak (EAdi_PEAK_), pressure time product of the trans-diaphragmatic pressure per breath and per minute (PTP_DI/b_ and PTP_DI/min_, respectively), breathing pattern and major asynchronies were continuously monitored for 12 h (from 8 a.m. to 8 p.m.). We identified breaths with “Normal” (EAdi_PEAK_ 5–15 μV), “Low” (EAdi_PEAK_ < 5 μV) and “High” (EAdi_PEAK_ > 15 μV) neuro-ventilatory drive.

**Results:**

Within all the analyzed breaths (177.117), the neuro-ventilatory drive, as expressed by the EAdi_PEAK_, was “Low” in 50.116 breath (28%), “Normal” in 88.419 breaths (50%) and “High” in 38.582 breaths (22%). The average times spent in “Low”, “Normal” and “High” class were 1.37, 3.67 and 0.55 h, respectively (*p* < 0.0001), with wide variations among patients. Eleven patients remained in the “Low” neuro-ventilatory drive class for more than 1 h, median 6.1 [3.9–8.5] h and 6 in the “High” neuro-ventilatory drive class, median 3.4 [2.2–7.8] h. The asynchrony index was significantly higher in the “Low” neuro-ventilatory class, mainly because of a higher number of missed efforts.

**Conclusions:**

We observed wide variations in EAdi amplitude and unevenly distributed “Low” and “High” neuro ventilatory drive periods during 12 h of PSV in critically ill patients. Further studies are needed to assess the possible clinical implications of our physiological findings.

## Introduction

Compared to controlled mechanical ventilation, mechanical assistance to spontaneous ventilation has the potential to improve gas exchange, hemodynamics, diaphragmatic function and comfort in most critically ill patients [1–3]. Pressure support ventilation (PSV) is the most used assisted mode of ventilation [4]. During PSV, the ventilator applies a constant (operator set) level of positive pressure throughout patient’s spontaneous inspiration and the inspiratory flow results from the interplay between patient’s inspiratory effort, assistance level and respiratory system impedance (mainly, resistance and elastance). Cycling to the expiratory phase occurs when the instantaneous inspiratory flow decays below a pre-definite threshold, usually an adjustable percentage of peak inspiratory flow [5]. Theoretically, PSV should support the respiratory muscles allowing spontaneous breathing with a “normal” neuro-ventilatory drive [6–8]. Over-assistance would result in low neuro-ventilatory drive putting the patient at risk of diaphragmatic atrophy [9, 10] while, on the other hand, under-assistance would result in high neuro-ventilatory drive, dyspnea [11], diaphragmatic fatigue and patient self-inflicted lung injury (P-SILI) [12]. Assessing the neuro-ventilatory drive would be pivotal to set and monitor PSV, but, unfortunately, is difficult to realize in clinical practice [13]. Accordingly, respiratory rate (RR), tidal volume (VT), patient-ventilator synchrony and gas exchange are taken into account to set PSV in the clinical setting [14–17]. To our knowledge, the neuro-ventilatory drive has not been continuously assessed during a prolonged period of PSV in critically ill patients.

The electrical diaphragmatic activity (EAdi) is the temporal sum of the electromyographic potentials of the crural diaphragm recorded by an array of electrodes mounted on the wall of a nasogastric tube [18, 19]. A dedicated software (Servo i, Getinge, Solna, SW) integrates and converts into a single amplitude/time signal the signals recorded by each electrode pair, taking into account the inspiratory displacement of the diaphragm [20]. The EAdi is a close surrogate of the neuro-ventilatory drive [13, 21–23] and is proportional to work of breathing [24].

In this study, we continuously assessed for 12 h the neuro-ventilatory drive (as expressed by the EAdi) in critically ill patients ventilated in the PSV mode. Due to different anatomical characteristics between patients, it is difficult to establish absolute reference EAdi values [25–27]. Nevertheless, in order to favor data analysis, based on previous studies [7, 25, 28, 29] we identified periods of “Normal” (EAdi_PEAK_ 5–15 μV), “Low” (EAdi_PEAK_ < 5 μV) and “High” (EAdi_PEAK_ > 15 μV) neuro-ventilatory drive. Our aim was to document the occurrence and entity of periods of “Low” and/or “High” neuro-ventilatory drive during clinical PSV application.

## Methods

### Patient selection

Patients admitted over a period of six months to the ICUs of the University of Bari and Ferrara Academic Hospitals were considered for enrollment in the study. The study was approved by the Ethics Committee of the Azienda Ospedaliero-Universitaria Policlinico di Bari (protocol no. 257) and of the Arcispedale Sant’Anna hospital, Ferrara, Italy (protocol no. 131084). Informed consent was obtained from each patient according to local regulations. The study was conducted between January 2016 and July 2016 in accordance with the Declaration of Helsinki. A physician not involved in the study was always present for patient care.

Patients were eligible for the study if they were older than 18 years and excluded if they were affected by neurological or neuromuscular pathologies, had known phrenic nerve dysfunction or any contraindication to the insertion of a naso-gastric catheter (for example: recent upper gastrointestinal surgery, esophageal varices).

### Measurements

Patients were studied in the semi-recumbent position. All the patients were ventilated with a Servo i ventilator (Maquet Critical Care, Solna, Sweden) equipped with the neurally adjusted ventilatory assist (NAVA) software that includes the “neuro-ventilatory tool” for EAdi measurement. At the beginning of the study, the standard naso-gastric tube was replaced with a 16 Fr, 125 cm, EAdi catheter (Maquet Critical Care, Solna, Sweden) unless an EAdi catheter was already in place. The EAdi catheter was first positioned according to the corrected nose-ear lobe-xyphoid distance formula and subsequently through the EAdi catheter position tool (Servo i, NAVA software) [30].

Peak airway opening pressure (P_AO PEAK_) and positive end-expiratory pressure (PEEP) were measured from the P_AO_ signal. Tidal volume (VT) was measured as the integral of the inspiratory flow. Mechanical respiratory rate (RR), inspiratory and expiratory time (Ti,_MECH_ and Te,_MECH_, respectively) were measured by the flow and P_AO_ signals. The inspiratory EAdi peak (EAdi_PEAK_), the slope of the EAdi from the beginning of inspiration to the peak (EAdi_SLOPE_) and the neural inspiratory time (Ti_NEUR_) were measured from the EAdi waveform.

The inspiratory pressure generated by the diaphragm (trans-diaphragmatic pressure, P_DI_) was calculated according to the method recently validated by Bellani and coworkers [7, 18, 21, 31]. Briefly, we first calculated the diaphragmatic neuro-muscular efficiency (NME) as the ratio between the negative deflection peak in P_AO_ during a spontaneous inspiratory effort (recorded during a brief end-expiratory occlusion lasting 5–10 s) and the corresponding peak in the EAdi curve. The NME measures the diaphragmatic neuro-mechanical coupling, and can be used to convert the EAdi into P_DI_ (P_DI_ = EAdi * NME) [31, 32]. The inspiratory P_DI_ pressure–time product per breath (PTP_DI_/b) was calculated as the area under the P_DI_ signal. The inspiratory P_DI_ pressure–time product per minute (PTP_DI_/min) was calculated as:

$${\text{PTP}}_{{{\text{DI}}}} /{\text{min}} = {\text{PTP}}_{{{\text{DI}}}} /{\text{b}} * {\text{RR}}.$$

The breathing pattern and EAdi-parameters, obtained from the RS232 port of the Servo i ventilator at a sampling rate of 100 Hz, were stored in a personal computer (NAVA tracker software, Maquet Critical Care, Solna, Sweden) for subsequent analysis (ICU Lab automatic analysis software, Kleistek Engineering; Bari, Italy).

### Study protocol

According to our institutional clinical protocol, patients were switched from controlled ventilation to the PSV mode as soon as possible in their clinical course, despite they were not deemed ready to be weaned, in order to improve patient-ventilator synchrony and comfort, decrease the need of sedation, improve the hemodynamic profile and preserve the diaphragmatic function [1, 3]. The shift from the controlled to the PSV mode was performed as soon as the following criteria were satisfied: (a) improvement in the condition leading to acute respiratory failure; (b) ability to trigger the ventilator, i.e., to decrease pressure airway opening (PAO) > 3 cmH_2_O during a brief (5–10 s) end-expiratory occlusion test; PaO_2_ ≥ 60 mmHg or SpO_2_ ≥ 90% on FiO_2_ ≤ 0.60 and PEEP ≤ 15 cm H_2_O c) Richmond Agitation Sedation Scale (RASS) between 0 and − 1 [33] with no sedation or a continuous infusion of dexmedetomidine (0.1–1.4 μg/kg/h); (d) hemodynamic stability without vasopressor or inotropes (excluding a dobutamine and dopamine infusion lower than 5 gamma/kg/min and a 3 gamma/kg/min, respectively) and normothermia.

Patients were admitted to the study within 24 h after the shift in the PSV mode. At the beginning of the study, the PSV level was carefully titrated to obtain a VT between 5 and 8 ml/kg predicted body weight (PBW) and a RR between 20 and 30 breaths/min [6, 34, 35]. The inspiratory trigger was set in the flow-by mode, sensitivity level 5 (Servo i arbitrary units); the expiratory trigger was set at 30% of the peak inspiratory flow. Clinical PEEP and FiO_2_ levels were left unchanged. Starting from the end of the PSV titration phase, patients were studied for 12 h, from 8 a.m. to 8 pm.

Throughout the study, the attending physicians could shift the patients from PSV to another ventilator mode, in the presence of any of the following conditions: a) need of a PSV level > 20 cmH_2_O or a PSV level + PEEP > 30 cmH_2_O, dyspnea, diaphoresis, paradoxical breathing, use of accessory respiratory muscles, need for neuromuscular blockade and/or deep sedation, hypoxemia (defined as a PaO_2_ ≤ 60 mmHg or SpO_2_ ≤ 90% or need of a FiO_2_ ≥ 0.60) or hypercapnia (pH lower than 7.35 for respiratory causes), hemodynamic instability.

### Data analysis

In order to continuously assess the neuro-ventilatory drive throughout the 12-h study period, the EAdi waveforms were analyzed through the automatic EAdi analysis software, a dedicated function of the ICU Lab software (Kleistek Engineering; Bari, Italy). This software identifies the EAdi peaks corresponding to each single breath and transfers the EAdi-related data (EAdi_PEAK_, EAdi_SLOPE_, Ti_NEUR_) in an excel sheet. Since the breathing pattern parameters (VT, RR, Pao_PEAK_, Ti_MECH_) could not be examined continuously by the software, for each patient we analyzed manually the first 30 consecutive breaths available for each neuro-ventilatory drive class through the dedicated function of the Kleistek software.

Based on previous studies [7, 25, 28, 29] and on the manufacturer instructions (Maquet Critical Care AB, NAVA flow chart MX-6462 Rev 02/2015), we pre-defined three neuro-ventilatory drive classes: “Low”, for breaths with EAdi_PEAK_ below 5 μV; “Normal”, for breaths with EAdi_PEAK_ between 5 and 15 μV and “High” for breaths with EAdi_PEAK_ higher than 15 μV.

Patient-ventilator asynchronies were assessed by taking into account in the first 20 consecutive min, for each EAdi class, based on the method proposed by Thille and coworkers [36]. Asynchronies were classified into six types: (a) ineffective triggering (missed effort); (b) ineffective inspiratory triggering; (c) double-triggering; (d) auto-triggering; (e) prolonged cycle; (f) short cycle [36]. The Asynchrony Index (AI) was calculated as:

$${\text{AI}} = {\text{Total number of asynchronies}}/({\text{mechanical cycles}} + {\text{missed efforts}}).$$

### Statistical analysis

We assessed the number the percentage of time spent in each of the three pre-defined EAdi_PEAK_ classes (i.e., “Low”, “Normal” and “High”). Differences between percentages were analyzed through the chi-square test. In order to estimate the average time spent in each of the three EAdi classes, we applied the Generalized Estimated Equation (GEE) model [37]. In the GEE model, the single breath is the first level unit, the time of each breath is the dependent variable, the class of EAdi is the independent variable and, finally, the patient is the second-level unit. Pairwise comparisons between the estimate times spent in each of the three neuro-ventilatory drive classes were adjusted according to Tukey.

Normally distributed continuous data are expressed as means and standard deviation (SD) and non-normally distributed data are expressed as median and interquartile range (IQR). Normality of continuous data was tested through the Kolmogorov–Smirnov test. The ANOVA or the Friedman-repeated measure analysis of variance was used as appropriate. Pairwise comparisons were adjusted according to Tukey.

A multivariable multinomial logistic model for ordinal variables and repeated measures was applied to evaluate the effect of Ti_MECH_, Pao_PEAK_, VT/PBW, RR and PS level on the probability of being in one of the three EAdi_PEAK_ classes. All the statistical tests were two-tailed, and *p-*values of less than 0.05 were considered statistically significant. Statistical analysis was performed by software SAS 9.4 (SAS Institute, Cary NC).

## Results

**Fig. 1:**
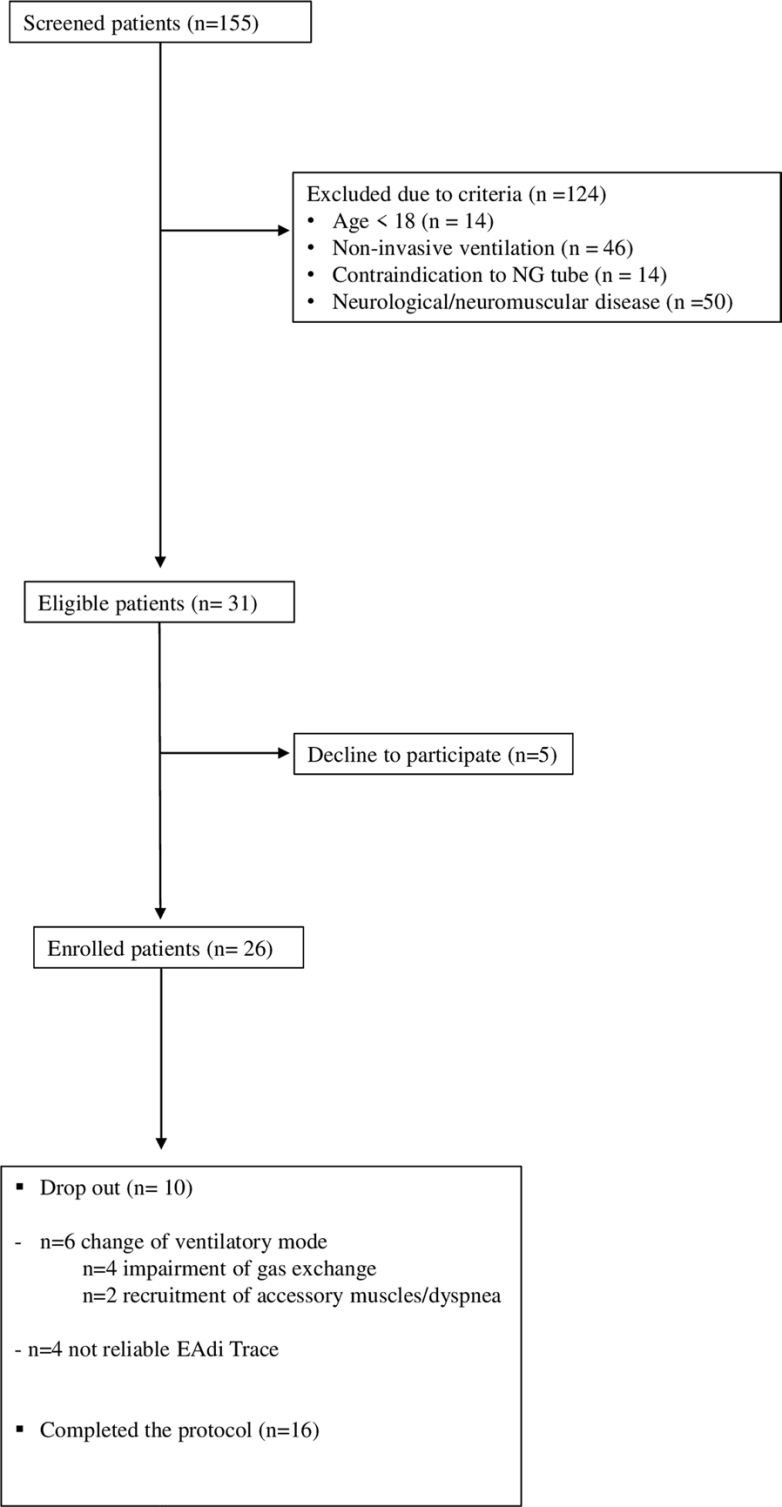
Flow diagram of patient’s enrollment. NG = naso-gastric; EAdi = electric diaphragmatic activity

Of the 155 patients admitted in the study period, 31 were eligible to the study. Five of them declined to participate and 26 were enrolled. Ten were dropped out from the study: in 6 the ventilation mode was changed during the 12-h study period and in 4 the EAdi trace was not reliable (Fig. 1). The demographical and clinical characteristics of the 16 studied patients are shown in Table 1.

**Table 1:** Baseline demographic and clinical characteristics of the patients

Pt #	Age	Sex	PBW (Kg)	SAPS II	Causes of ARF	MV (Days)	PSV level (cmH_2_O)	PEEP (cmH_2_O)	FiO_2_%	RASS	NME (cmH_2_O/µV)	ICU outcome
1	70	M	70	63	Septic shock	10	8	13	40	0	0.62	Alive
2	70	M	67	35	Acute post-operative respiratory failure	11	10	7	40	− 1	1.66	Alive
3	71	M	66	39	Trauma	13	8	8	50	0	1.58	Dead
4	71	M	53	39	Septic shock	2	10	6	50	0	1.92	Alive
5	69	F	60	49	Thoracic Injury	2	13	7	40	0	1.76	Dead
6	70	M	68	31	Hemorrhagic stroke	8	10	8	40	− 1	2.41	Alive
7	85	M	71	40	Drowning	5	13	7	35	− 1	1.01	Dead
8	75	F	53	43	Septic shock	7	8	5	40	− 1	0.9	Alive
9	59	M	57	27	Hemorrhagic shock	12	9	10	30	− 1	1.74	Alive
10	61	M	53	33	Septic shock	11	9	10	50	− 1	3.2	Alive
11	78	M	79	46	COPD exacerbation	5	14	5	50	0	3.45	Alive
12	84	M	67	30	Community acquired pneumonia	4	10	5	40	0	1.89	Alive
13	49	M	64	36	Septic shock	11	11	10	30	− 1	2.52	Alive
14	73	F	48	38	Acute post-operative respiratory failure	8	10	5	40	0	3.77	Alive
15	80	M	66	40	COPD exacerbation	11	9	8	50	−1	0.98	Dead
16	70	M	70	45	Septic shock	7	18	10	50	0	2.05	Alive
Mean	70.18		63.25	39.12		7.9	10.65	7.76	43.24	− 0.5	1.96	
SD	9.38		8.4	8.64		3.5	2.60	2.28	8.09	0.52	0.9	

PBW = predicted body weight; SAPS II = Simplified Acute Physiology score II. The score can range from 0 to 163, with higher scores indicating a higher probability of death; ARF = acute respiratory failure; MV = mechanical ventilation; PSV level = pressure support ventilation level; PEEP = positive end-expiratory pressure; FiO_2_ = inspiratory oxygen fraction; RASS = Richmond Agitation Sedation Scale; ICU = intensive care unit; COPD = chronic obstructive pulmonary disease

**Fig. 2:**
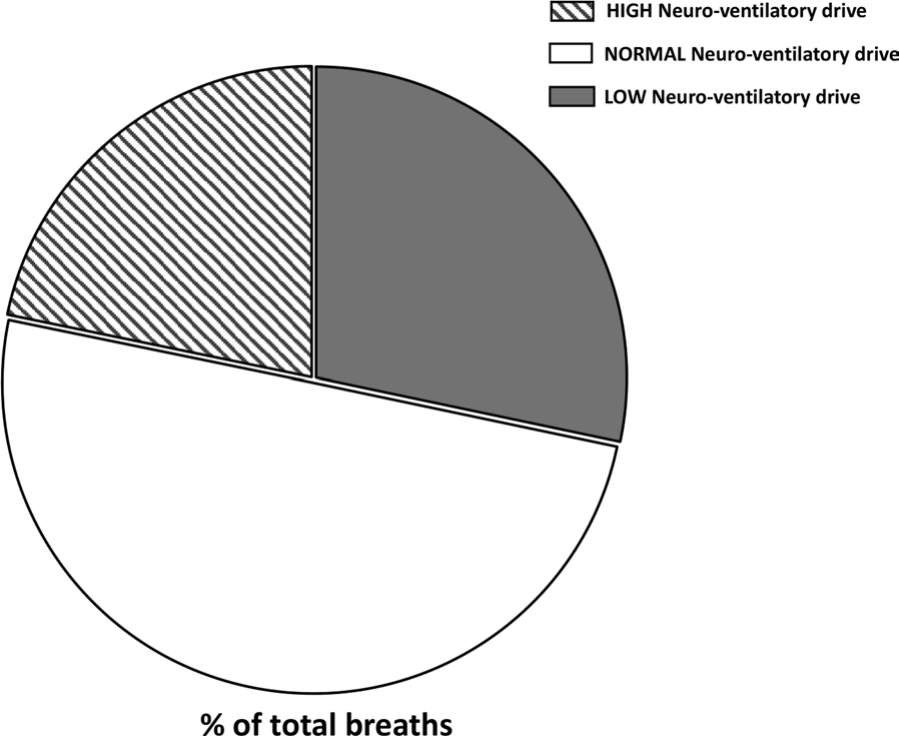
Percentage of the collected breaths (including all the patients and the whole study period) belonging to the “Low” (28%), “Normal” (50%) and “High” (22%) neuro-ventilatory drive class (chi-square = 45; *p* = 0.0001)

Taking all the patients as a whole, 177.117 breaths were collected throughout the study period. The neuro-ventilatory drive was “Low” in 50.116 breaths (28%), “Normal” in 88.419 breaths (50%) and “High” in 38.582 breaths (22%) (chi-square = 45; *p* = 0.0001) (Fig. 2). The GEE model showed a significant difference between the time spent in the three different EAdi classes and estimated an average time of 1.37 h for the “Low”, 3.67 h for the “Normal” and 0.55 h for the “High” class (*p* < 0.0001). The difference between the average time spent in the "Normal" and "High" class was statistically significant (*p* = 0.019). The intra-patient variability (± standard error) was 0.025 ± 0.104, while the residual variability was 0.908 ± 0.192. Accordingly, the intra-class correlation coefficient was low (2.7%) and not statistically significant, indicating that, among the patients, the time spent in each neuro-ventilatory drive class was heterogenous.

**Fig. 3:**
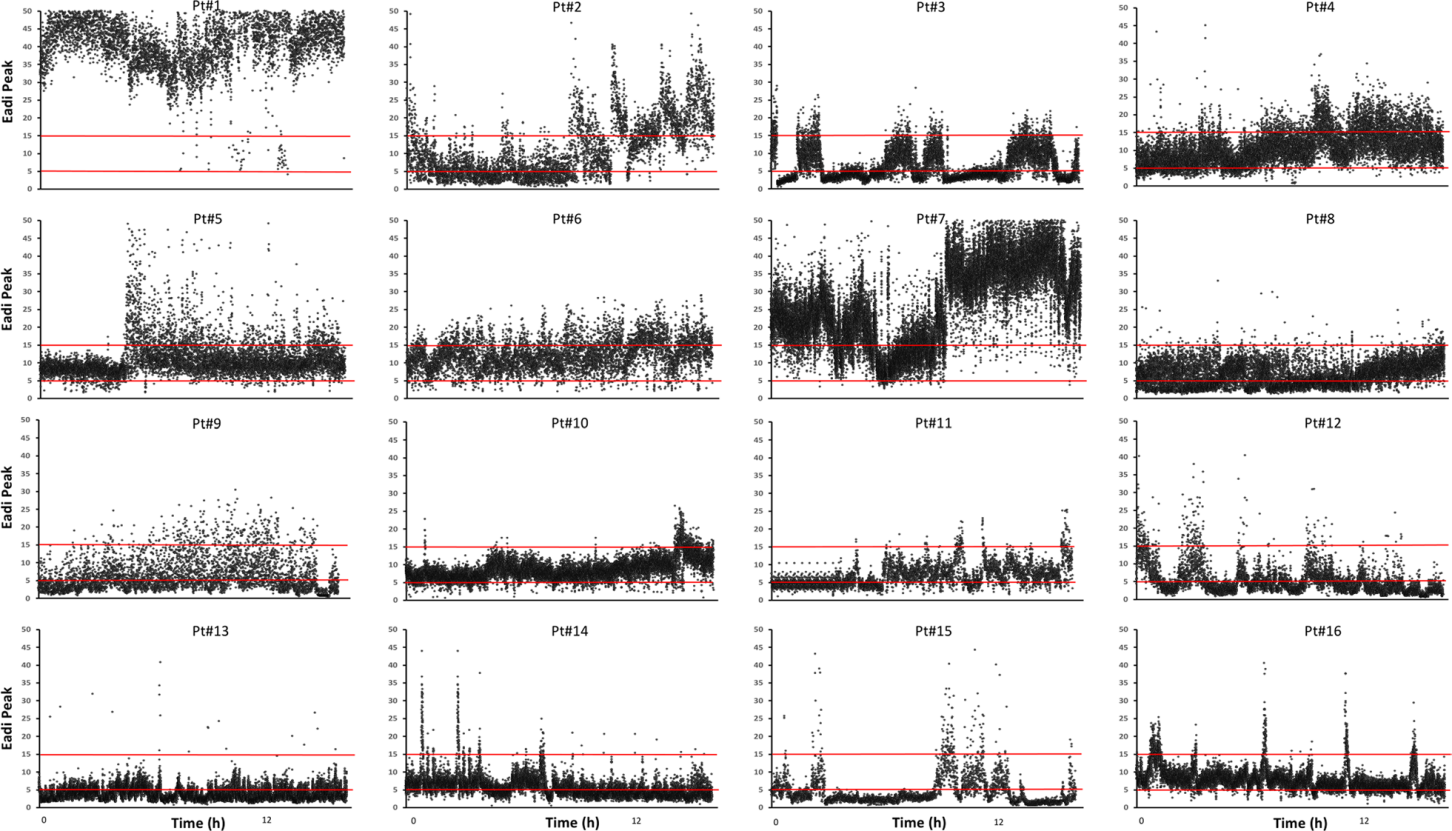
Individual neuro-ventilatory drive trend throughout the study. Each point represents the electric diaphragmatic activity peak (EAdi_PEAK_) of a single breath. The two red lines represent the 5–15 μV EAdi_PEAK_ range depicting the “Normal” neuro-ventilatory drive

**Fig. 4:**
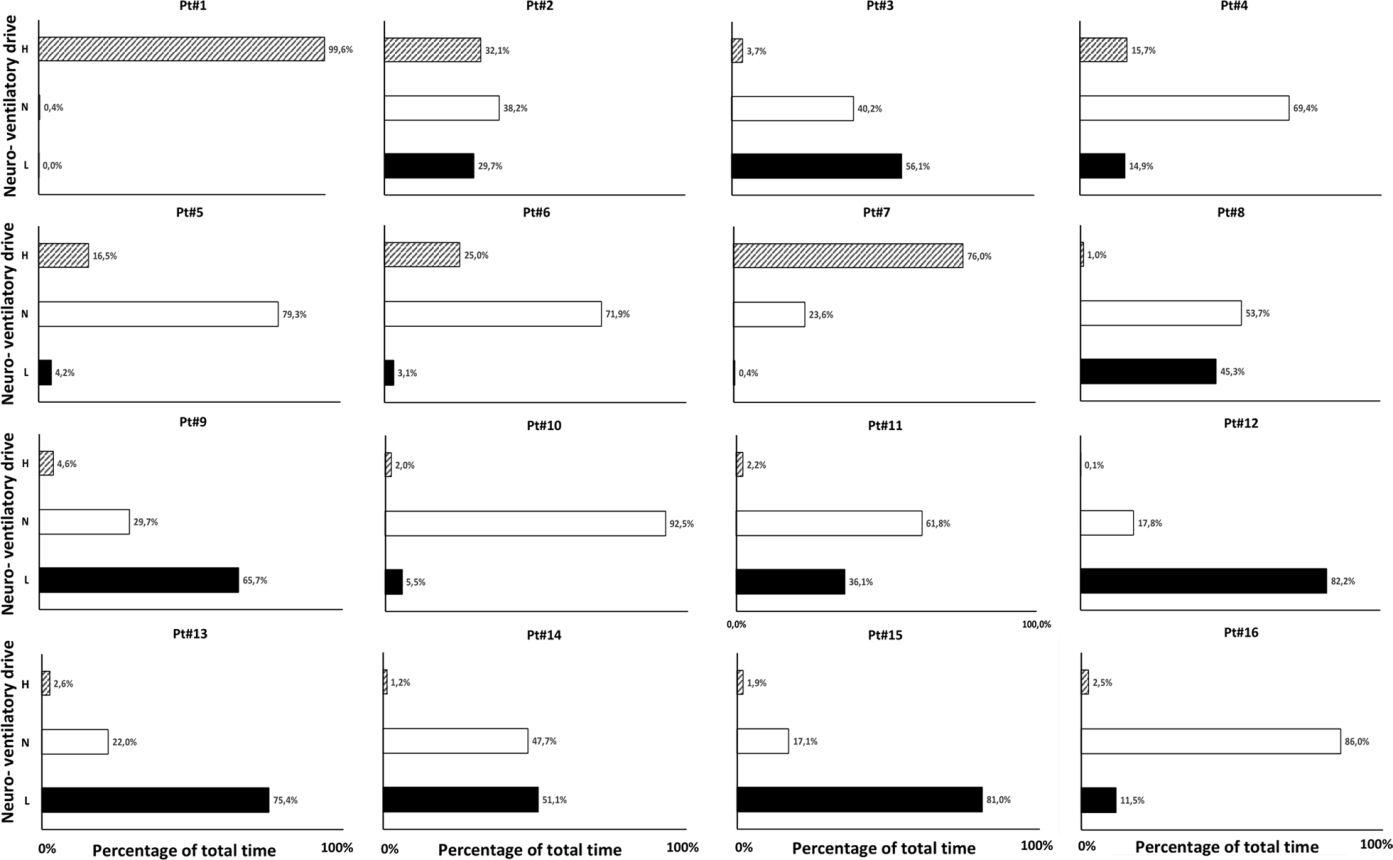
Individual percentage of study time in which each patient remained in the “Low”, “Normal” and “High” neuro-ventilatory drive class. Eleven patients remained in the “Low” neuro-ventilatory drive class for more than one hour, median 6.1 [3.9–8.5] h. Six patients remained in the “High” neuro-ventilatory drive class for more than one hour, median 3.4 [2.2–7.8] h

Figures 3 and 4 show, respectively, the individual trend of the neuro-ventilatory drive throughout the study and the percentage of study time in which each patient remained in the “Low”, “Normal” and “High” neuro-ventilatory drive class. Eleven patients remained in the “Low” neuro-ventilatory drive class for more than one hour, median 6.1 [3.9–8.5] h. Six patients remained in the “High” neuro-ventilatory drive class for more than one hour, median 3.4 [2.2–7.8] h.

Table 2 reports the breathing pattern, EAdi and work of breathing parameters referred to the three neuro-ventilatory drive classes. Compared to the “Low” class, tidal volume was significantly higher in the “Normal” and “High” neuro-ventilatory drive class. Both PTP_DI_/b and PTP_DI_/min significantly increased going from the “Low” to the “High” class.

**Table 2:** Physiological parameters, referred to the three EAdi-defined neuro-ventilatory drive classes

	“Low” class (EAdi_PEAK_ < 5 μV)	“Normal” class (EAdi_PEAK_ 5–15 μV)	“High” class (EAdi_PEAK_ > 15 μV)
VT/PBW (ml/kg)	7.2 [6.2–8.3]	7.5 [6.3–9.2]*	8.8 [6.9–9.5]*
RR (breaths/min)	19.7 [15.0–28.9]	19.1 [15.8–22.7]*	15.6 [14.8–21.7]*^#^
PEEP (cmH_2_O)	6.2 [5.2–9.9]	7.2 [5.9–8.0]	7.8 [6.7–12.1]
Pao,_PEAK_ (cmH_2_O)	17.5 [14.4–20.9]	18.0 [16.8–18.7]	19.3 [18.0–20.0]*^#^
Ti,_MECH_ (s)	0.97 [0.76–1.18]	1.02 [0.86–1.17]	1.01 [0.80–1.14]*^#^
Ti,_NEUR_ (s)	1.05 [0.81–1.27]	1.17 [0.93–1.41]	1.13 [0.83–1.38]*^#^
EAdi_PEAK_ (μV)	3.2 [2.1–4.1]	8.0 [6.5–10.1]	19.9 [17.2–28.5]*^#^
EAdi_SLOPE_ ( μV/s)	2.6 [1.9–3.7]	6.4 [5.1–9.3]*	21.1 [14.7–27.8]*^#^
PTP_DI_/b (cmH_2_O/s)	2.2 [1.4–3.4]	6.6 [3.8–9.3]*	12.4 [8.0–17.3]*^#^
PTP_DI_/min (cmH_2_O/s/min)	46 [29–76]	110 [73–164]*	213 [160–309]*^#^

Data expressed as median and interquartile range [IQR]

EAdi = diaphragmatic electrical activity; VT = tidal volume; PBW = predicted body weight; RR = respiratory rate; PEEP = positive end expiratory pressure; Pao,_PEAK_ = peak airway opening pressure; Ti,_MECH_ = mechanical inspiratory time; Ti,_NEUR_ = neural inspiratory time; EAdi_PEAK_ = peak diaphragmatic electrical activity; EAdi_SLOPE_ = slope from the beginning of inspiration to EAdi_PEAK_; PTP_DI_/b = inspiratory pressure–time product of the diaphragm, per breath; PTP_DI_/min = inspiratory pressure–time product of the diaphragm, per minute

^*^p < 0.05 compared to the “Low” EAdi class

^#^*p* < 0.05 compared to the “Normal” EAdi class

Table 3 shows the major patient-ventilator asynchronies in the three classes. The asynchrony index was significantly higher in the “Low” neuro-ventilatory class, mainly because of a higher number of missed efforts.

**Table 3:** Main asynchronies and asynchrony index

	“Low” class (EAdi_PEAK_ < 5 μV)	“Normal” class (EAdi_PEAK_ 5–15 μV)	“High” class (EAdi_PEAK_ > 15 μV
Missed efforts (*n*/min)	0.12 ± 0.04	0.05 ± 0.04*	0.07 ± 0.05*^#^
Ineffective inspiratory triggering (*n*/min)	0.05 ± 0.007	0.02 ± 0.03	0.03 ± 0.03
Double triggering (*n*/min)	0.007 ± 0.004	0.01 ± 0.006	0.003 ± 0.002
Prolonged cycles (*n*/min)	0.005 ± 0.004	0.00 ± 0.002	0.003 ± 0.003
Short cycles (*n*/min)	0.00 ± 0.00	0.00 ± 0.002	0.00 ± 0.001
Asynchrony index (%)	20.6 ± 3.5	10.1 ± 9.6*	12.3 ± 4.9*^#^

**Fig. 5:**
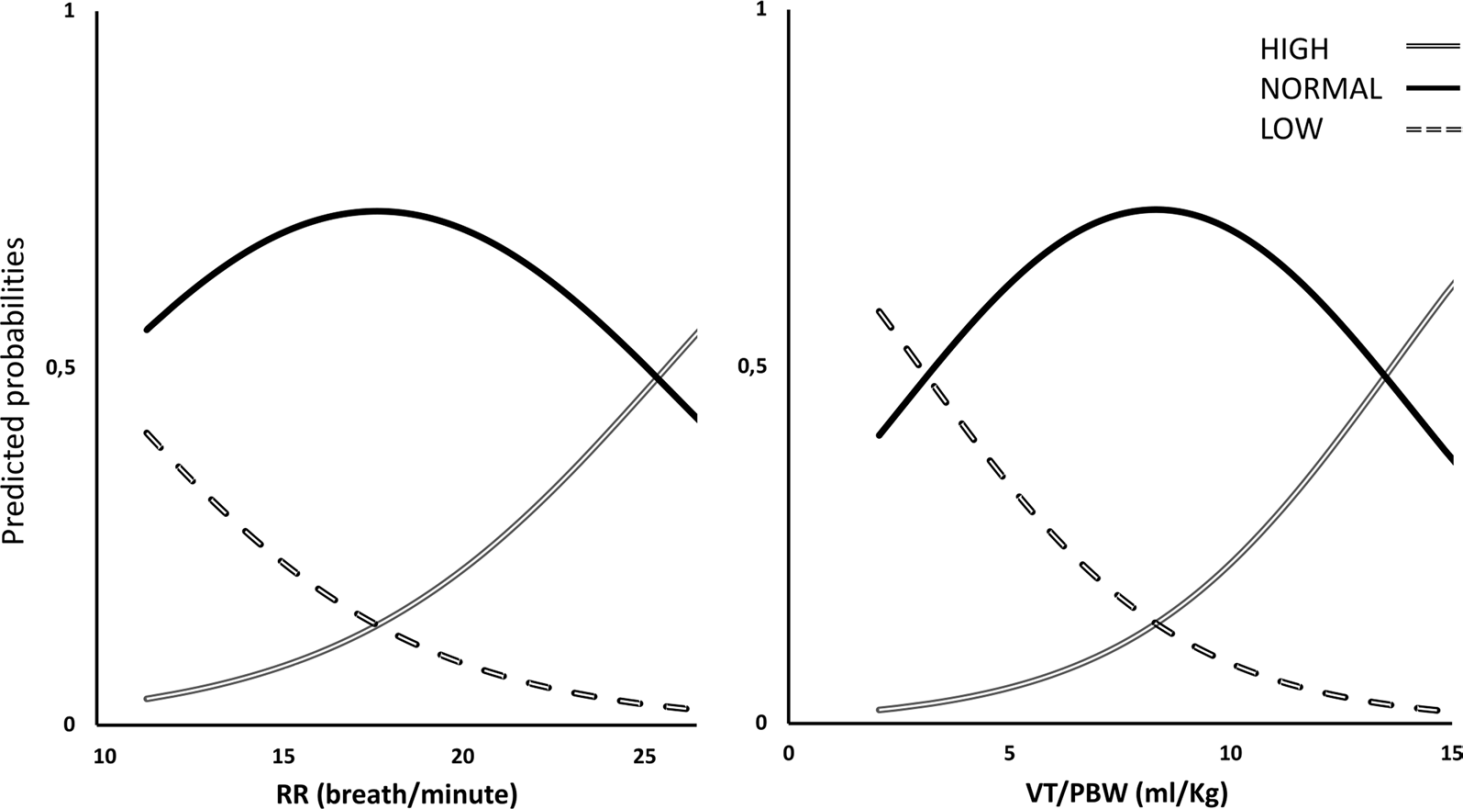
According to the multivariable multinomial logistic model, the risk of being in the “High” neuro-ventilatory drive class increased exponentially with respiratory rate (RR, Left Panel) and tidal volume/predicted body weight (VT/PBW, Right Panel)

Figure 5 shows that, according to the multivariable multinomial logistic model, the risk of being in the “High” neuro-ventilatory drive class increased exponentially with VT (Panel A) and with RR (Panel B). The odds ratio of being in a neuro-ventilatory drive class different than “Normal” increased by 1.41 [CI 95%, 1.16–1.70], *p* = 0.0019, for each deviation of 1 ml/kg PBW from the median VT (7.4 ml/kg PBW) and by 1.1 [CI 95%, 1.0–1.24], *p* = 0.0472, for each deviation of 1 breath/min from the median RR (18.5 breaths/min). Tinsp_MECH_, Pao_PEAK_ and the PSV level were not included in the model because not significant at the univariate analysis.

## Discussion

By monitoring for 12 h the EAdi in critically ill patients ventilated in the PSV mode, we observed unevenly distributed periods of “Low” and/or “High” neuro-ventilatory drive.

By amplifying the patient’s breathing effort, mechanical assistance should normalize the neuro-ventilatory drive when the respiratory muscles are challenged by an absolute or relative increase in workload [16, 38, 39]. However, the PSV algorithm leaves to the clinician the task of setting the level of assistance and, therefore, of estimating patient’s work of breathing and neuro-ventilatory drive [17, 40, 41]. Physiological observations during stepwise PSV titration suggest that excessive or insufficient assistance (over and under-assistance, respectively) are associated with peculiar breathing patterns. Briefly, low VTs (i.e., lower than 5 ml/kg PBW) and high RRs (i.e., higher than 30 breaths/min) denote under-assistance, whereas high VTs (i.e., higher than 8 ml/kg PBW) and low RRs (i.e., lower than 20 breaths/min) denote over-assistance [17, 42, 43]. In this study, according to these physiological observations and to a consolidated clinical protocol [6, 34, 44], we titrated PSV to a VT between 5 and 8 ml/PBW and a RR between 20 and 30 breaths/min. The fact that we found wide variations in neuro-ventilatory drive since the beginning of the study challenges the “classical” approach to PSV setting (Fig. 3). Despite our study was physiologically oriented and conducted in a small cohort of patients, we believe that these findings could be of clinical interest for studies aiming at defining more physiological protocols for setting the assistance level during PSV.

As reviewed elsewhere [8, 11, 26], the neuro-ventilatory drive originates from the respiratory centers, a network of interconnected neurons in the pons and medulla and is modulated by gas exchange, physical exercise, sleep, emotional and behavioral inputs, pain, discomfort, sedation and analgesia. In pathological conditions, air trapping, decreased lung and/or chest wall compliance, increased airway resistance and/or respiratory muscle weakness may alter the coupling between patient’s effort and diaphragmatic excursion (neuro-ventilatory coupling), increasing the neuro-ventilatory drive [16, 45–47]. In our patients, we observed wide variations in neuro-ventilatory drive despite the sedation level was kept constant throughout the study period (i.e., RASS score between 0 and − 1) [33]. To explain these findings, one can hypothesize that our patients underwent to subclinical episodes of discomfort or pain, able to increase the neuro-ventilatory drive, or, on the contrary to excess of sedation or sleep able to decrease the neuro-ventilatory drive. Indeed, sleep rhythm and architecture are disrupted in critically ill patients [48–50]. These variations in neuro-ventilatory drive would have been “primary”, i.e., independent from the ventilatory assistance. Another possible hypothesis, however, is that the metabolic status and/or the mechanical workload posed on the respiratory muscles varied during the study and the PSV mode was not able to appropriately “satisfy” the changing patient’s requirements. In the latter case, it would have been appropriate to classify the “Low” and “High” neuro-ventilatory drive periods as over or under-assistance, respectively [38]. Unfortunately, our study design does not allow to outline if the neuro-ventilatory drive varied for a primary or a secondary mechanism and, accordingly, we are not able to classify as over- or under-assistance the “Low” or “High” neuro-ventilatory drive episodes recorded in our patients.

It is worth remarking that during PSV the assistance level is fixed and it has been shown that the response to a sudden metabolic [51] or elastic load [16] is “not physiologically oriented” (i.e., rapid shallow breathing). At variance with PSV, during the neurally adjusted ventilatory assist mode (NAVA) the assistance is proportional to the EAdi [19, 52], during the proportional assist ventilation *plus* mode (PAV) the assistance is proportional to the patient’s inspiratory effort [38, 39, 53] and during the adaptive support mode (ASV) the ventilator adapts the assistance according to a closed-loop algorithm to optimize the WOB through the Otis Equation [54, 55]. Thus, an attracting hypothesis is that the “proportional” modes or the ASV would stabilize the neuro-ventilatory drive more than PSV [28, 56].

During PSV, a “Low” neuro-ventilatory drive puts the patients at risk of diaphragmatic atrophy and patient-ventilator asynchrony [9, 10, 57]. Interestingly, we found an asynchrony index of 20.6 ± 3.5% during the “Low” neuro-ventilatory drive periods (Table 3), well above the 10% threshold, that predicts prolonged weaning and ICU length of stay [36, 58]. On the other hand, an “High” neuro-ventilatory drive may induce diaphragmatic disfunction and patient self-inflicted lung injury [13, 59–61]. Previous data from our group suggest that a prolonged PSV period (48 h) does not improve diaphragmatic efficiency [7]. Based on the present data, we speculate that the concurrence of “Low” and “High” neuro-ventilatory drive periods could explain our previous findings.

Overall, our physiological data support the idea that the neuro-ventilatory drive should be continuously monitored during PSV in critically ill patients. The EAdi tool may represent a reasonable approach, since the EAdi is a “close” peripheral surrogate of the neuro-ventilatory drive, but there are several drawbacks that must hamper any over-enthusiasm. First, the relation between EAdi amplitude and breathing effort is not linear but depends from the neuro-ventilatory coupling [38]. For example, in case of diaphragmatic atrophy, the EAdi signal may be detectable in the absence of detectable pneumatic breathing efforts [62]. Second, the EAdi tool is commercially available in one ventilator only. Third, in several patients it is technically difficult or even impossible to obtain a reliable EAdi monitoring, even in experienced hands [7]. In the present study, we were not able to obtain a readable EAdi signal in 4 out of 26 patients (15.4%), despite our groups are experienced in clinical EAdi monitoring (Fig. 1). This suggests caution when trusting on the EAdi monitoring in the clinical context. According to the expert’s opinion, monitoring the breathing effort through esophageal manometry or estimating it through diaphragmatic echography could be an alternative [13, 26, 27, 42]. However, it should be kept in mind that EAdi amplitude and breathing effort convey different information [25].

We must acknowledge the following study limitations. First***:*** the EAdi_PEAK_ thresholds applied in the present study to classify the neuro-ventilatory drive are empirical, although based on previous studies [7, 25, 28, 29] and on the manufacturer’s instructions (Maquet Critical Care AB, NAVA flow chart MX-6462 Rev 02/2015). Indeed, the EAdi signal is burdened by interindividual variability [63]. Nevertheless, we identified a rather broad “Normal” EAdi_PEAK_ range similar to the one recently observed by Piquilloud and coworkers in healthy volunteers supported with different level of PSV [25] and by Liu and coworkers in patients [64]. Another important issue in favor of our approach is that in our patients the median PTP_DI_/min (a parameter of work of breathing) was 110 [73–164] cmH_2_O/s/min in the “Normal” EAdi class (Table 2), largely within the normal PTP_DI_/min range identified by classical physiological studies, i.e., between 50 and 150 cmH_2_O/s/min [65]. On the other hand, despite the absolute EAdi thresholds are debatable, our data clearly document a wide EAdi variability over time in otherwise clinically stable patients during PSV (Fig. 3), and therefore, despite the absolute EAdi thresholds remain debatable, our study effectively quantified the neuro-ventilatory drive trend during PSV *Second:* an important issue to interpret our results is how the PSV level was set. As discussed above, our intent was to reproduce the “real life” in the clinical scenario [66–68]. However, we cannot exclude that different approaches to PSV setting would have a different impact on the neuro-ventilatory drive [28]. *Third:* ours was a physiologically oriented study and thus we have no data on the impact of the neuro-ventilatory drive patterns on diaphragmatic and pulmonary functions.

## Conclusions

We observed wide variations in EAdi amplitude and unevenly distributed “Low” and “High” neuro-ventilatory drive periods during 12 h of PSV in critically ill patients. Further studies are needed to assess the possible clinical implications of these physiological findings.

## Data Availability

The study dataset used is available upon a justified request.

## Abbreviations

PSV: Pressure support ventilation
EAdi: Electrical activity of the diaphragm
EAdi_PEAK_: Peak EAdi
PTP_DI_/b: Pressure–time product of the P_DI_ per breath
PTP_DI_/min: Pressure–time product of the P_DI_ per minute
P-SILI: Patient self-induced lung injury
RR: Respiratory rate
VT: Tidal volume
NAVA: Neurally adjusted ventilatory assist
P_AO_: Pressure airway opening
P_AO PEAK_: Peak airway opening pressure
PEEP: Positive end-expiratory pressure
Ti,_MECH_: Mechanical inspiratory time
Te,_MECH_: Mechanical expiratory time
Ti,_NEUR_: Neural inspiratory time
NME: Neuro-muscular efficiency
RASS: Richmond agitation sedation scale
PBW: Predicted body weight
FiO_2_: Oxygen inspiratory fraction
AI: Asynchrony Index
GEE: Generalized estimated equation
PAV: Proportional assist ventilation
